# Mapping interfacial hydration in ETS-family transcription factor complexes with DNA: a chimeric approach

**DOI:** 10.1093/nar/gky894

**Published:** 2018-10-08

**Authors:** Amanda V Albrecht, Hye Mi Kim, Gregory M K Poon

**Affiliations:** 1 Department of Chemistry, Georgia State University, Atlanta, GA 30303, USA; 2 Center for Diagnostics and Therapeutics, Georgia State University, Atlanta, GA 30303, USA

## Abstract

Hydration of interfaces is a major determinant of target specificity in protein/DNA interactions. Interfacial hydration is a highly variable feature in DNA recognition by ETS transcription factors and functionally relates to cellular responses to osmotic stress. To understand how hydration is mediated in the conserved ETS/DNA binding interface, secondary structures comprising the DNA contact surface of the strongly hydrated ETS member PU.1 were substituted, one at a time, with corresponding elements from its sparsely hydrated relative Ets-1. The resultant PU.1/Ets-1 chimeras exhibited variably reduced sensitivity to osmotic pressure, indicative of a distributed pattern of interfacial hydration in wildt-ype PU.1. With the exception of the recognition helix H3, the chimeras retained substantially high affinities. Ets-1 residues could therefore offset the loss of favorable hydration contributions in PU.1 via low-water interactions, but at the cost of decreased selectivity at base positions flanking the 5′-GGA-3′ core consensus. Substitutions within H3 alone, which contacts the core consensus, impaired binding affinity and PU.1 transactivation in accordance with the evolutionary separation of the chimeric residues involved. The combined biophysical, bioinformatics and functional data therefore supports hydration as an evolved specificity determinant that endows PU.1 with more stringent sequence selection over its ancestral relative Ets-1.

## INTRODUCTION

Water strongly mediates the affinity and specificity of protein/DNA interfaces (1,2). Hydration contributions to binding arise from differences in molecular interactions available to water in the bulk medium relative to those in the environs of the macromolecules. At protein/DNA interfaces, water may be excluded, interact dynamically with the interface or form structured bridges that effectively become part of the complex. The influence of interfacial hydration on protein/DNA complexes is thus complex, but critical for understanding the molecular basis of target selection. For example, restriction endonucleases distinguish sequence-similar DNA substrates based in part on differences in the interfacial hydration of the enzyme/DNA complex (3–6). During catalysis, the disposition of water changes as the protein/DNA interface shifts from a substrate-binding conformation to one that is poised for reaction of the transition state (7,8). As a result of substrate-dependent hydration contributions, restriction enzymes discriminate ‘star’ sequences that they will cleave from non-specific unreactive sequences even though the binding affinities for both DNA are essentially the same (9).

While transcription factors lack catalytic activity, hydration plays similarly important roles in their target DNA recognition. The *Escherichia coli* tryptophan repressor is iconic in this respect (10), and both the crystal structure (11) as well as experiments in solution (12) show that it recognizes operator DNA primarily via water-mediated contacts. More recently, we have found that hydration is strongly coupled with site discrimination by the ETS-family protein PU.1, a master transcription factor in hematopoiesis (13). Typical of this family of transcription factors, PU.1 recognizes cognate DNA sites ∼10 bp in length that contain a 5′-GGA(A/T)-3′ central consensus. Variants of this motif in which the core consensus is flanked by different sequences bind PU.1 with a broad range of dissociation constants from 10^−10^ to 10^−7^ M under physiologic conditions (14). This *in vitro* selectivity correlates closely with PU.1 cognate sites in native target promoters and enhancers (15).

When probed by osmotic pressure, high-affinity binding by PU.1 is profoundly destabilized due to the accumulation of hydration water in the protein/DNA interface (16). In addition, binding to low-affinity cognate sites is distinguishable from non-specific binding in terms of their hydration properties. Low-affinity cognate binding is not osmotically sensitive, while non-specific binding is strongly stabilized by osmotic pressure, even though the apparent affinities of the two modes differ only marginally in the absence of osmotic stress. Thus hydration distinguishes target recognition by PU.1, which regulates a large genetic network in hematopoietic cells (17), of cognate versus non-specific binding sites as well as optimal versus low-affinity cognate sites.

The sequence-specific effects of hydration in PU.1/DNA binding prompted us to ask whether they represented a class property of the ETS family, a major lineage of metazoan transcription factors. ETS proteins are united by their DNA-binding domains, known as ETS domains, which are strongly homologous in structure (18). They share overlapping DNA preferences that match sequence motifs bound by their full-length parents (19). Despite these similarities, lineage-restricted ETS proteins such as PU.1 are functionally non-redundant and only partially interchangeable with close phylogenetic relatives *in vivo* (20,21). Molecular mechanisms that confer functional specificity to ETS proteins therefore represent a focal point of their molecular biology in development and disease (22). In particular, general mechanisms that govern the DNA-binding behavior of ETS domains are of fundamental biological interest.

In this connection, we reported that Ets-1, a structural homolog that is co-expressed with PU.1 during hematopoiesis (23,24), exhibits markedly different DNA-binding properties with respect to interfacial hydration. Although the ETS domains of PU.1 and Ets-1 are divergent in amino acid sequence, they fold into superimposable structures and bind their optimal DNA targets with similarly high affinity as PU.1 in the absence of osmotic stress. However, unlike PU.1, Ets-1 is minimally sensitive to osmotic pressure regardless of DNA sequence (25). As a functional consequence, we noticed that the two ETS relatives also differ in their levels of site selectivity. Analysis of DNA sequence motifs for the two homologs by information theory shows that PU.1 is significantly more selective in target readout than Ets-1 (26). The higher stringency by PU.1 is fully maintained in data taken from *in vitro* site selection assays using recombinant ETS domains, as well as ChIP-Seq experiments probing native proteins in human and mouse cells (26). Thus, PU.1 is more target-selective than Ets-1 *in vivo*, and this stringency is accrued from intrinsic differences in DNA recognition between their ETS domains.

In addition to serving as an experimental probe for studying hydration changes, osmotic stress is a physiologic condition in hematopoietic tissues (27,28). We analyzed gene expression data in murine macrophages and found that target genes for PU.1 are significantly over-represented in osmotically responsive genes (25). Recently, *hypo*-osmotic stress in K562 leukemic cells was reported to *drive* PU.1 binding to promoter sites in significant excess over Ets-1 (29), in agreement with their osmotic profiles in binding experiments. The differential interfacial hydration in DNA binding therefore also appears to establish the two ETS members’ roles in cellular response to physiologic osmotic stress.

The biological significance of hydration in DNA recognition and gene regulation by PU.1 and Ets-1 highlights a need to better understand the physical basis of their hydration differences. Recently, we probed the structure of interfacial hydration in optimal PU.1/DNA complexes by mutating a conserved water-coordinating Tyr residue in PU.1 to Phe (30). This one-atom difference reduced PU.1 sensitivity to osmotic pressure by ∼25% without affecting binding affinity under normo-osmotic conditions. This unexpected observation suggested that hydration of the PU.1/DNA interface might not be essential to binding, and alternative interactions could compensate for reduced hydration contributions to binding affinity.

To resolve the structural basis of hydration in the PU.1/DNA interface and characterize the effects of hydration on binding affinity and selectivity, we used a chimeric approach to map the hydration landscape in the PU.1/DNA interface. Specifically, we took advantage of the strong structural conservation among ETS domains and asked whether the hydration contributions within the DNA contact interface could be mapped to specific secondary structures comprising that surface. We evaluated PU.1 mutants in which secondary structures comprising the DNA contact surface were replaced one at a time by the corresponding element from Ets-1. Perturbations in the DNA binding affinity of these PU.1/Ets-1 chimeras and their osmotic sensitivity would provide insight into the contribution of the substituted element to interfacial hydration as well as the attendant effects on target affinity and selectivity.

## MATERIALS AND METHODS

### Molecular cloning

Codon-optimized DNA fragments encoding various PU.1 mutants were synthesized by IDT DNA Technologies (Midland, IA, USA) and subcloned into NcoI/HindIII sites of pET28b vector for overexpression in *E. coli*, or into NheI/HindIII sites of pcDNA3.1(+) for eukaryotic expression. The bacterial constructs harbored a C-terminal cleavage site for thrombin followed by a 6xHis tag. All constructs were verified by Sanger sequencing.

### Protein expression and purification

Heterologous overexpression of wild-type (WT) (PU.1ΔN167) and chimeric PU.1 ETS domains in BL21(DE3)pLysS *E. coli* was performed as previously described (31). In brief, expression cultures in LB media were induced at an OD_600_ of 0.6 with 0.5 mM isopropyl β-D-1-thiogalactopyranoside for 4 h at 25°C. Harvested cells were lysed by sonication in 0.1 M TrisHCl, pH 7.4 containing 0.5 M NaCl and 5 mM imidazole. After centrifugation, cleared lysate was extracted with Co-NTA and eluted in up to 15 ml of elution buffer containing 150 mM imidazole. The eluate was dialyzed overnight in the presence of 10 U of thrombin (MPBio) against 10 mM NaH_2_PO_4_/Na_2_HPO_4_, pH 7.4 containing 0.5 M NaCl and polished on Sepharose SP (HiTrap, GE). After extensive washing in this buffer, the protein was eluted in a NaCl gradient at ∼1 M. Purified protein was dialyzed extensively into various buffers appropriate for the experiments, and diluted as needed with dialysate. Protein concentrations were determined by UV absorption at 280 nm. Each construct was verified by MALDI-ToF(+) analysis (Supplementary Table S1).

### Circular dichroism spectroscopy

Far-UV (190–250 nm) spectra were acquired in 10 mM NaH_2_PO4/Na_2_HPO_4_, pH 7.4, and 0.15 M NaCl with a Jasco J-810 instrument.

### Osmotic stress experiments

DNA binding experiments by fluorescence anisotropy measurements of a Cy3-labeled DNA probe were performed as described (30,32,33). Briefly, sub-saturating concentrations of WT or chimeric PU.1 (10^−9^ to 10^−8^ M) were incubated to equilibrium with graded concentrations of an unlabeled 23-bp DNA duplex oligo harboring the high-affinity PU.1 target site 5′-AGCGGAAGTG-3′ or the low-affinity site 5′-AAAGGAATGG-3′. The binding mixtures contained 10 mM TrisHCl, pH 7.4, 0.15 M NaCl, 0.1 mg/ml bovine serum albumin, and various concentrations of betaine to achieve the desired osmotic pressure. Solution osmolality was determined by vapor pressure measurements on a Wescor VAPRO 5600 instrument. Steady-state anisotropies $\langle r \rangle$ were measured at 595 nm in 384-well black plates (Corning) in a Molecular Dynamics Paradigm reader with 530 nm excitation. The signal represented the fractional bound DNA probe (*F*_b_), scaled by the limiting anisotropies of the bound $\langle {{r_1}} \rangle$ and unbound states $\langle {{r_0}} \rangle$ as follows:

(1) \begin{equation*}\left\langle r \right\rangle = {F_{\rm{b}}}\left( {\left\langle {{r_1}} \right\rangle - \left\langle {{r_0}} \right\rangle } \right) + \left\langle {{r_0}} \right\rangle \end{equation*}

*F*_b_ as a function of *total* unlabeled DNA concentration was fitted to a mutually exclusive binding model as detailed previously (32) and summarized in Supplementary Methods for convenience.

### Molecular dynamics simulations

Explicit-solvent simulations were performed with the Amber14SB/parmbsc1 forcefields (34) in the GROMACS 2016.4 environment. Homology models of WT and chimeric PU.1ΔN167 in complex with the 23-bp experimental DNA sequence were generated against the co-crystal PU.1/DNA complex (PDB ID: 1PUE; 35) as a template using the SWISS-MODEL server (36). The starting models were solvated with TIP3P water and 0.15 M NaCl in a dodecahedral box whose edge was at least 1.0 nm from the closest atom in the model. All simulations were carried out at an *in silico* temperature and pressure of 298 K (modified Berendsen thermostat) and 1 bar (Parrinello-Rahman ensemble). All bonds were constrained using LINCS. After the structures were energy-minimized by steepest descent, the NVT ensemble was equilibrated at 298 K for 100 ps to thermalize the system followed by another 100 ps of equilibration of the NPT ensemble at 1 bar and 298 K. Production simulation was performed for 400 ns with configurations saved every 10 ps for analysis. Convergence of the trajectories was checked by RMSD from the energy-minimized structures. Average structures were taken as the middle snapshot determined by a Jarvis-Patrick clustering procedure based on the mutual RMSD of the all snapshots from the final 100 ns of the simulations.

### Cellular reporter assays

Cellular PU.1 transactivation was measured using a PU.1-dependent EGFP reporter construct as previously described (37). In brief, the EGFP reporter is under the control of a minimal PU.1-dependent enhancer harboring a 5 × tandem of a native cognate site for PU.1. In PU.1-negative HEK293 cells, the reporter was transactivated in the presence of an expression plasmid encoding WT full-length PU.1 and a co-translating iRFP marker (38). 7 × 10^4^ cells were seeded in 24-well plates and co-transfected with a cocktail consisting of the EGFP reporter plasmid (300 ng) and expression plasmids for full-length PU.1 (50 ng) and WT/mutant ETS domains (150 ng), using JetPrime reagent (Polyplus, Illkirch, France) according to the manufacturer’s instructions. One or both of the expression plasmids were replaced by empty pcDNA3.1(+) vector in negative control samples. Eighteen hours after transfection, the cells were trypsinized and analyzed by flow cytometry using a FCS Fortessa instrument (BD). Live cells were gated for iRFP and EGFP fluorescence using reporter- and full-length PU.1-only controls, respectively, in FlowJo (BD) before computing the total fluorescence of the dually fluorescent population. Expression of the ETS domains were quantified with ImageJ software from immunoblots of cell lysate after probing with a polyclonal anti-PU.1 antibody (GenScript A01692) with GAPDH as loading control.

## RESULTS

**Figure 1.**
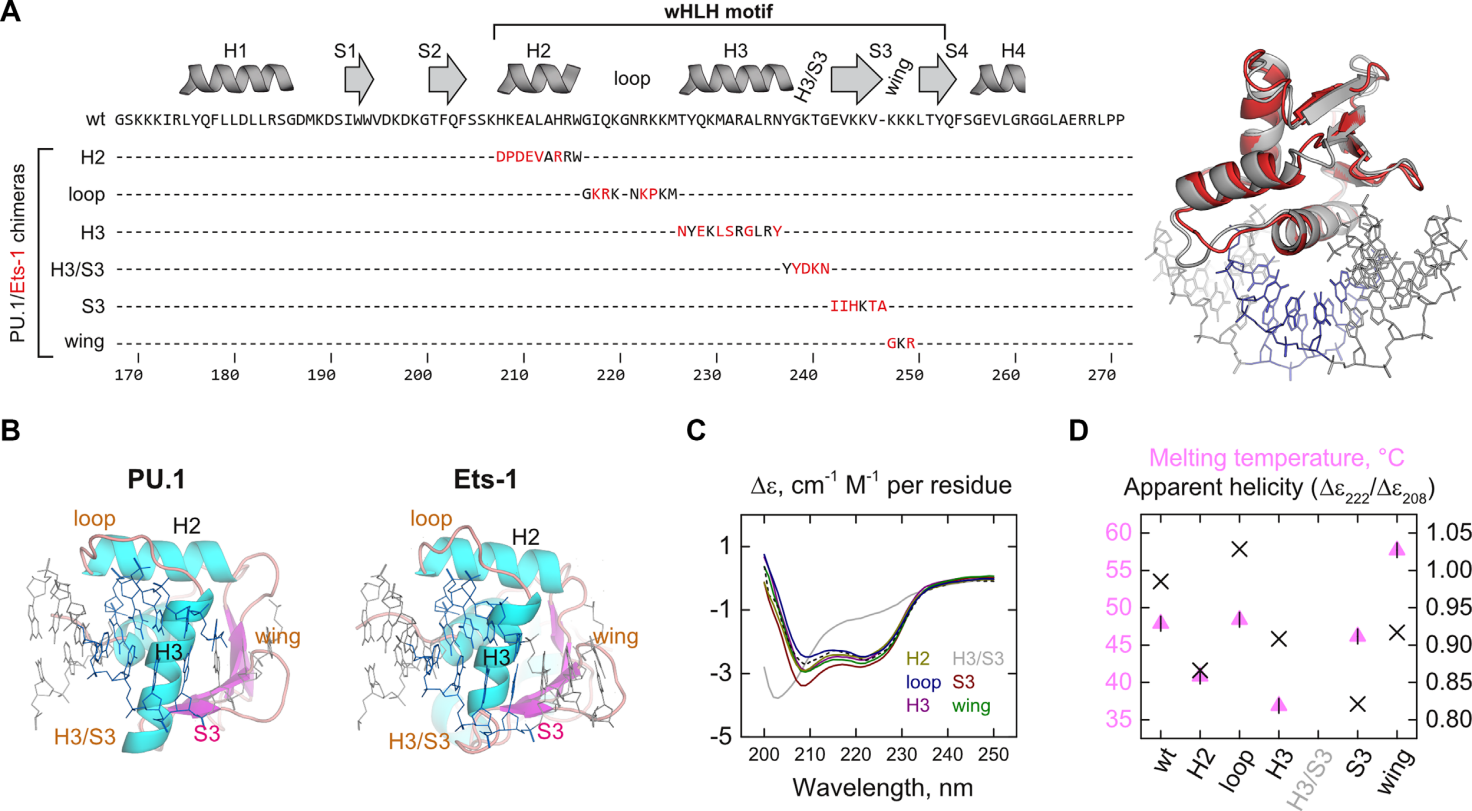
A chimeric approach to mapping PU.1/DNA interfacial hydration. (**A**) Amino acid sequences of the six PU.1/Ets-1 chimeras. Each of the secondary structure elements that comprise the winged helix-loop-helix DNA-binding motif in PU.1 is replaced with the corresponding element in Ets-1. WT PU.1 and Ets-1 are superimposable in their DNA-bound states (PDB IDs: 1PUE and 1K79). (**B**) Topology of DNA contact interfaces in WT PU.1 and Ets-1. Note the secondary structure assignments of H3/S3 in PU.1 versus Ets-1 as per the crystal structures (PDB IDs: 1PUE and 1K79). (**C**) Far-UV CD spectra of WT and chimeric PU.1 ETS domains at 25°C. (**D**) Melting temperatures of WT and chimeric PU.1 (triangles), plotted with an index for α-helical content taken as the ratio of the molar residue ellipticities at 222/208 nm.

Our chimeric approach to dissecting the interfacial hydration in the site-specific PU.1/DNA complex is shown in Figure 1. The chimeras were designed from a sequence alignment of murine ETS domains (Supplementary Figure S1) with a view of maintaining the register of residues that are most conserved among ETS paralogs. The secondary structural elements that comprise the DNA contact surface in order of primary structure are H2, loop, H3, H3/S3, S3, wing and S4, the last of which we had previously examined (30) (Figure 1A). These elements together comprise the conserved ‘winged helix-loop-helix’ motif (Figure 1B). H3 is the groove-binding recognition helix that interacts with the conserved 5′-GGA(A/T)-3′ core consensus in ETS binding sites. The extended loop and short wing connect H2 and H3, and S3 and S4, respectively. The H3/S3 segment is a sharp turn between H3 and S3 that includes several residues assigned as part of H3 in the PU.1/DNA complex (35). Here, we follow the assignment specified by the Ets-1/DNA co-crystal structure (39).

We first addressed whether the chimeras folded correctly relative to WT PU.1 by circular dichroism spectroscopy. Five out of the six chimeras exhibited similar secondary structure as WT PU.1, with strong α-helical content at 25°C (Figure 1C). The lone exception, H3/S3, appeared to be substantially unfolded. Thermal experiments showed that the five well-folded chimeras melted reversibly with melting temperatures (*T*_m_) no more than 10°C different from WT (Supplementary Figure S2). The variations in *T*_m_ did not correlate with secondary structure contents (Figure 1D) and were similar to published ETS mutants (30). Thus, with the exception of H3/S3, the PU.1/Ets-1 chimeras were natively folded under our experimental conditions as WT PU.1. These five chimeras were subsequently interrogated for their DNA-binding properties under osmotic pressure.

To determine the perturbation of the chimeric substitutions on DNA recognition, we measured the binding affinity of the chimeras for an optimal PU.1-binding sequence under osmotically variable conditions. This sequence is also bound by WT Ets-1 with high affinity (Supplementary Figure S3). Previously, we have extensively characterized the effect of compatible osmolytes on DNA binding by WT PU.1 and Ets-1 and found that they acted quantitatively in a colligative manner (i.e. independent of osmolyte identity), consistent with perturbing specific protein/DNA binding through osmotic pressure (16,25). The apparent structural conservation of WT PU.1, Ets-1 and their chimeras (except H3/S3) supports the utility of osmotic stress to probe changes in the latter’s interfacial hydration with DNA. Here, we used betaine, a widely used compatible osmolyte (40), to exert osmotic pressure.

**Figure 2.**
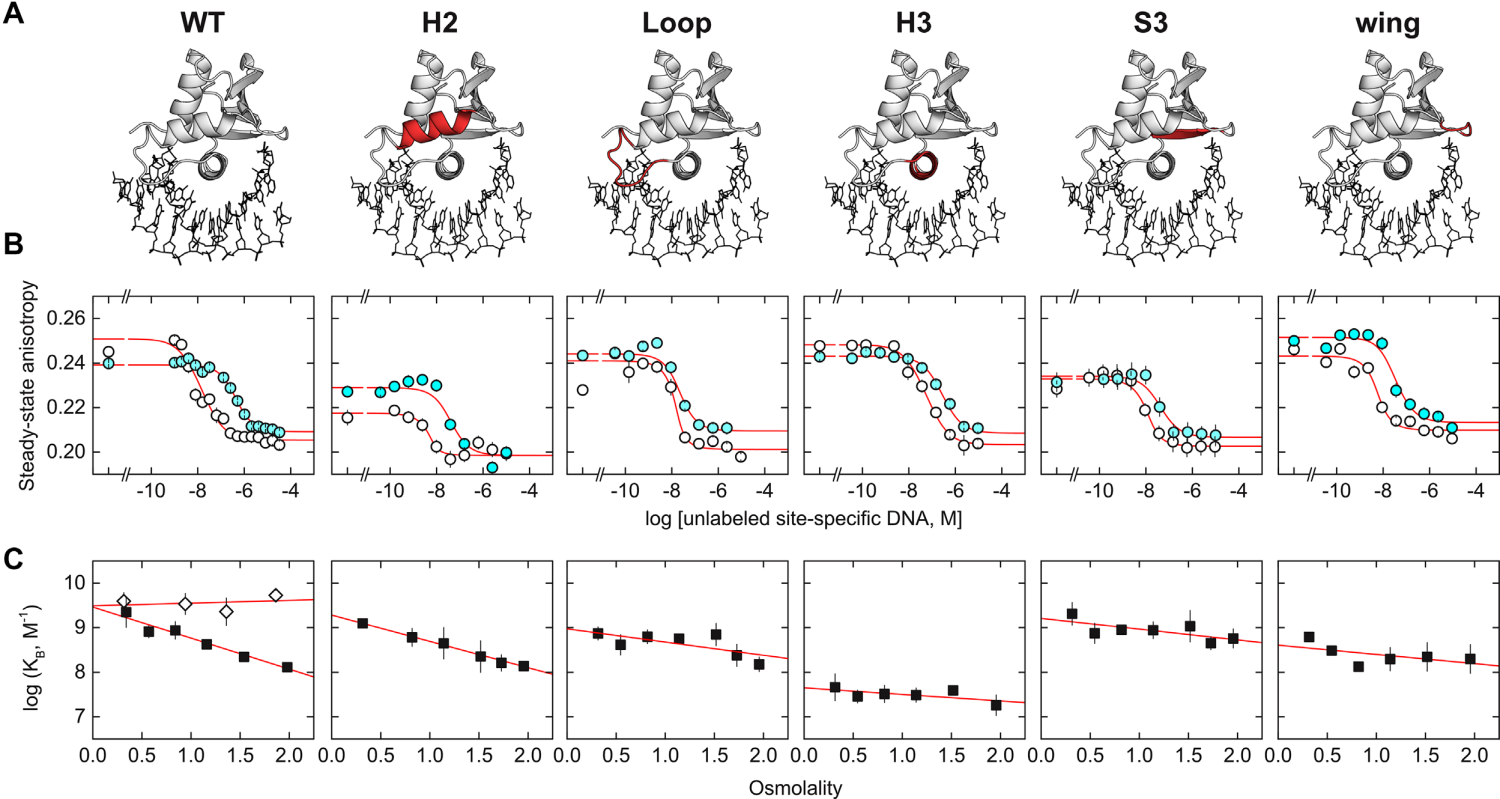
PU.1/Ets-1 chimeras reveal a dispersion of osmotic sensitivity and normo-osmotic DNA binding affinities. (**A**) Schematic showing the Ets-1 substitutions shaded. (**B**) Representative competitive titrations under normo-osmotic conditions (0.29 osm, open circles) and at 2 osm (solid). Increasing concentrations of unlabeled-specific DNA competitor displaces protein from a Cy3-labeled DNA probe, lowering its anisotropy. *Curves* represent fits of Equation (1) to the data. Variations in the limiting anisotropies and curvatures in the data arise from differences in affinity of the proteins to the probe and unlabeled DNA, protein concentrations used to acquire a sufficient change in anisotropy, betaine’s effects on the photophysical properties of the probe and small variations associated with plate-based detection. These variations are handled by the fitting model and frees the experimenter to select protein concentrations that balance the change in anisotropy with depletion of the titrant, given the wide range of affinities involved. The model directly estimates the affinity of the protein to the unlabeled target DNA, the parameter of interest (not IC_50_); see Supplementary Methods for details. (**C**) Osmotic pressure dependence of the binding affinity. Each point represents the average of three or more replicates ± S.D. WT Ets-1 is included as reference (diamonds). *Lines* represent linear fits to the data; parametric values of the slope (osmotic sensitivity, Γ) and normo-osmotic affinity (at 0.3 osm) are given in Table 1.

By differentially raising the free energy cost of assembling water of hydration in an interface that excludes osmolyte, osmotic pressure imposes a penalty on high-affinity DNA binding by WT PU.1. As a result, the logarithm of the binding constant for PU.1 decreases linearly by 1.5 orders of magnitude over 2 osmolal above normo-osmotic conditions (41). This slope is related to the net uptake or release of water molecules when strongly excluded osmolytes such as betaine are used to exert this pressure (42):

(2) \begin{equation*}\Gamma \equiv \frac{{\partial \log {K_{\rm{B}}}}}{{\partial \, {\rm{osm}}}} = - \frac{{\Delta {n_{\rm{w}}}}}{{55.5\ln 10}}\end{equation*}

where 55.5 × ln 10 = 128 and a positive value of $\Delta {n_{\rm{w}}}$indicates net uptake. When probed under WT identical solution conditions, all PU.1/Ets-1 chimeras exhibited the same linear trend but with variable slopes that were intermediate of WT PU.1 and Ets-1 (Figure 2 and Table 1). These shallower slopes indicated that the chimeric complexes retained intermediate levels of interfacial hydration and confirmed these chimeras as functioning hydration probes of the PU.1/DNA interface. In addition to the slope, the DNA-binding affinities at normo-osmotic pressure (0.3 osm) indicated whether the chimeric Ets-1 residues supported high-affinity binding in the PU.1 scaffold. Together, these two parameters describe the hydration contribution (slope) of a substituted element and whether chimeric Ets-1 residues confer alternative interactions that offset the loss of favorable hydration contributions (affinity at normo-osmotic pressure). Since the elements differ in size and exposure to the bound DNA, we also estimated their per-residue DNA contact surface area based on the WT PU.1/DNA complex to facilitate assessment of their binding properties.

**Table 1.** Binding properties of WT and chimeric PU.1 ETS domains

	Normo-osmotic affinity *K*_D_ × 10^−9^, M	Osmotic sensitivity Γ, osm^−1^	Hydration change $\Delta {n_{\rm{w}}}$	Contact area with DNA, Å²/residue
*WT*	0.34 ± 0.06	−0.70 ± 0.05	90 ± 6	21 ± 1 (overall)
*Chimeras*				
H2	0.80 ± 0.05	−0.59 ± 0.01	76 ± 1	4.0 ± 0.1
Loop	1.3 ± 0.4	−0.30 ± 0.14	39 ± 18	23 ± 1
H3	22 ± 10	−0.15 ± 0.09	19 ± 11	37 ± 1
H3/S3	nd	nd	nd	3.7 ± 0.2
S3	0.49 ± 0.12	−0.24 ± 0.10	31 ± 13	9.7 ± 0.2
Wing	1.6 ± 0.1	−0.21 ± 0.15	27 ± 19	30 ± 1

**Figure 3.**
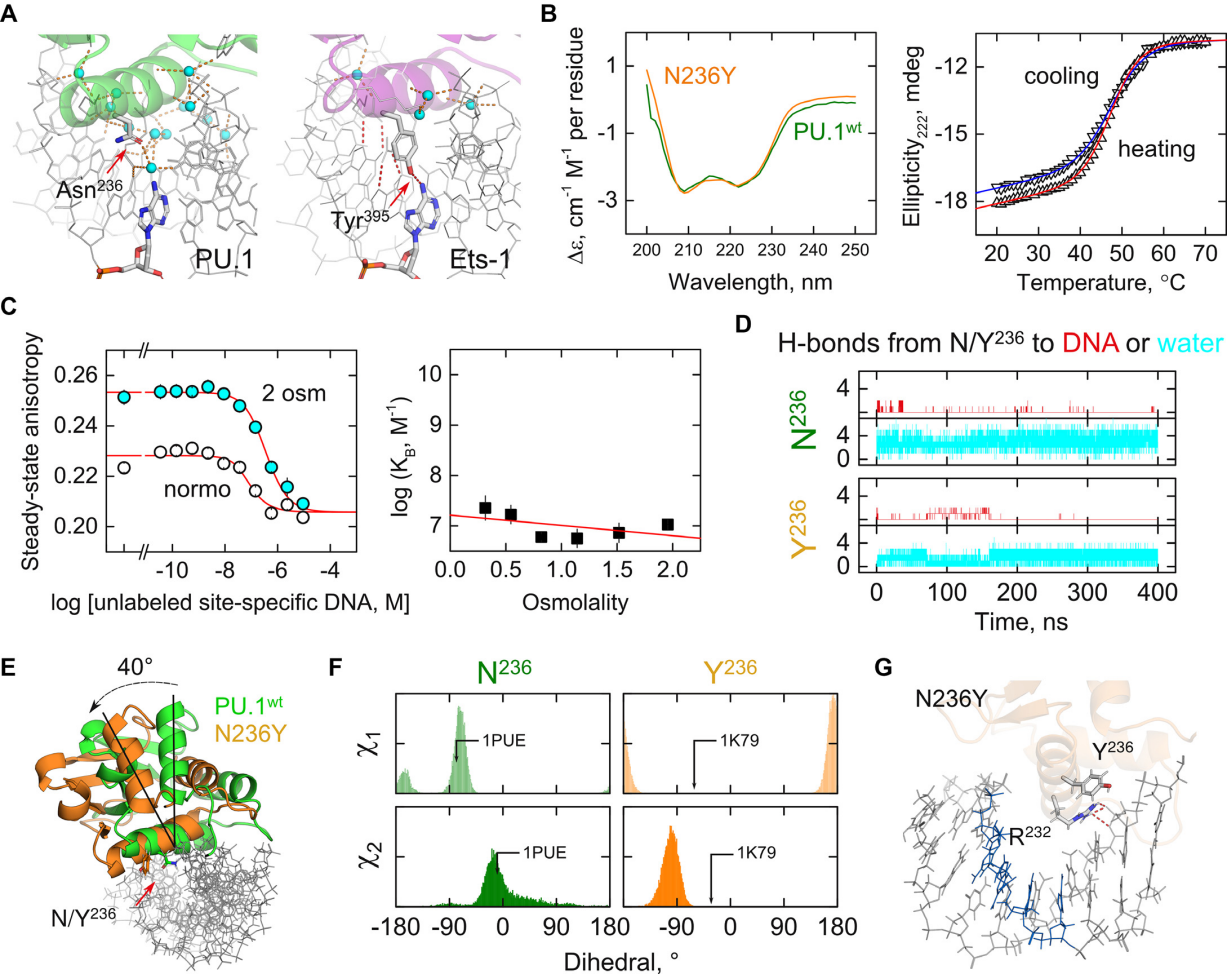
Asn^236^ in the recognition helix H3 is a keystone residue in DNA binding by PU.1. (**A**) Co-crystal structures of WT PU.1 and Ets-1 show contrasting roles for Asn^236^ in PU.1 and its Tyr^395^ counterpart in Ets-1. (**B**) The point chimera N236Y showed an identical CD spectrum at 25°C and reversible unfolding as WT PU.1. (**C**) Decompensated DNA binding by N236Y with loss of osmotic sensitivity. (**D**) All-atom MD simulation of WT and N236Y bound to the experimental DNA target. H-bonds (within 3.5 Å, ±30°) between Asn^236^ or Tyr^236^ and DNA or water molecules show an initial burst of direct DNA contacts by N236Y and partial desolvation of Tyr^236^ between 50 and 150 ns. (**E**) Alignment of the average structure from the final 100 ns shows a 40° re-orientation of the chimera relative to the DNA. (**F**) Distribution of the two standard sidechain dihedrals of Asn^236^ and Tyr^236^ in the final 100 ns. Arrows mark values from the co-crystal structures in Panel A. (**G**) Mapped average configurations of Tyr^236^ and Arg^232^ in H3 of N236Y, the latter of which has lost its absolutely conserved consensus contact (blue).

Site-specific affinity of WT and chimeric ETS domains of PU.1 was measured by competition by unlabeled target DNA against a Cy3-labeled DNA probe for protein. Representative titrations are shown in Figure 3B. Steady-state fluorescence anisotropy data were fitted with Equation (1). Averages ± SE of three to five independent replicates were used to determine the osmotic pressure dependence of the binding affinity as shown in Figure 2C. Normo-osmotic affinity is given as the equilibrium dissociation constant *K*_D_ (reciprocal binding constant). Osmotic sensitivity (Γ) is the linearly fitted slope of the osmotic pressure dependence. The implied change in hydration water molecules was determined from Γ via Equation (2). nd, not determined. The DNA contact area was computed from the WT PU.1/DNA complex (PDB ID: 1PUE) as the difference in solvent-accessible surface area of the structure in the presence and absence of the bound DNA (averaged for the two asymmetric units in the model).

The reduction in osmotic sensitivity among the chimeras tracked roughly with the per-residue DNA-contact area of the substituted element in WT PU.1 (Table 1). Thus, the H2 chimera, which is the least exposed to the DNA among the well-formed chimeras, also exhibited the smallest hydration perturbation among the tested chimeras. Conversely, the H3 and wing chimeras, which represent the most contacted elements in the interface, yielded the greatest losses in osmotic sensitivity. Chimeric substitution was more perturbative than expected at S3 based on DNA contact surface area, suggesting additional hydration contributions by this element above the others. Since destabilization by osmotic pressure reflects the favorable hydration contributions to binding, the data indicated that hydration was not localized to any one or small subset of secondary structures comprising the DNA contact surface in WT PU.1.

Binding affinity under normo-osmotic conditions also tracked roughly with osmotic sensitivity and DNA-contact area, but the perturbations were generally smaller in magnitude. Although chimeric substitutions of the H2, loop, S3 and wing of Ets-1 into a PU.1 significantly blunted sensitivity to osmotic pressure, binding affinity was reduced by no more than 5-fold at normo-osmotic pressure. This was not a significant change in affinity given the >400-fold span of affinities with which WT PU.1 binds cognate DNA sites (14). Thus, favorable hydration contributions in WT PU.1 to affinity were offset by alternative interactions by the Ets-1 residues in these chimeras. In the case of the loop, S3 and wing chimeras, the decoupling of hydration from high-affinity binding rendered their binding profiles similar to Ets-1, which binds optimal DNA with similar affinity as PU.1 but minimal osmotic sensitivity (c.f. Figure 2A).

### The H3 chimera is significantly impaired in both normo-osmotic binding and osmotic sensitivity

The H3 chimera differed from the other constructs in its significant loss of binding affinity (>50-fold) as well as osmotic sensitivity. This was a biologically significant level of reduction, within ∼10-fold of the affinity of WT PU.1 for low-affinity specific sites (14). Thus, unlike the other elements, Ets-1 residues in the recognition helix H3 were incompatible with high-affinity binding in a PU.1 scaffold.

To identify non-conserved residues responsible for the low tolerance to chimeric substitution in H3, we noticed that this helix is the most conserved element among ETS domains. Every H3 position is either fully or conservatively substituted, except residue 236. In WT PU.1, this residue is Asn, which is unique to PU.1 and its two closest ETS relatives (Supplementary Figure S1). In the WT PU.1/DNA complex, Asn^236^ is exclusively engaged in water-mediated contacts with the core consensus in the major groove. In contrast, the corresponding position in WT Ets-1 is Tyr^395^, which is engaged in a direct H-bond with the exocyclic N6 of 5′-GGA-3′ in the consensus (Figure 3A). To probe the significance of Asn^236^ in PU.1, we mutated it to Tyr. The resultant N236Y chimera was conformationally conserved with WT PU.1 (Figure 3B), indicating that this substitution was non-perturbative in the unbound protein. Nevertheless, binding by N236Y was identical to the H3 chimera (Figure 3C). Chimeric substitution of Asn^236^ alone was therefore sufficient to reproduce the binding profile of the full H3 chimera.

To understand the loss of high-affinity binding by N236Y in greater detail, we performed all-atom molecular dynamics (MD) simulations of the site-specific WT PU.1 and N236Y complexes using the co-crystal WT structure as template. Following ∼0.2 μs of equilibration in unconstrained production, the WT complex achieved a stable ensemble around the canonical ETS/DNA conformation. Integrity of the interfacial hydration in the WT complex was evaluated in terms of contact between Asn^236^ and DNA versus water. The strong hydration of Asn^236^ in preference over direct DNA contact demonstrated consistency of the MD results with experimental data on WT PU.1 (Figure 3D). In contrast, the N236Y chimera exhibited altogether different dynamics. During the equilibration period (from ∼50 to 150 ns), it made frequent direct contact with N6 of 5′-GGAA-3′ with attendant partial desolvation of the sidechain. Strikingly, it then underwent a major transition that abolished these direct DNA contacts. The average final structures (from a clustering procedure) revealed a large re-orientation of the chimeric protein with respect to the target DNA (Figure 3E). More precisely, the protein pivoted ∼40° near the midpoint of H3 in the major groove but without significant rearrangement of its tertiary structure. While the WT Asn^236^ sidechain ensemble effectively maintained the same dihedrals as in the co-crystal structure, the Tyr^236^ sidechain in N236Y had swung out of position in the new orientation and no longer pointed into the major groove (Figure 3F). Moreover, canonical interactions between Arg^232^ with nucleobases in the consensus, a conserved H3 contact engaged by all specific ETS/DNA complexes (including PU.1), were replaced by contacts with the DNA backbone just beyond the core consensus (Figure 3G).

### Chimeric perturbation at Asn^236^ is evolutionarily sensitive

**Figure 4.**
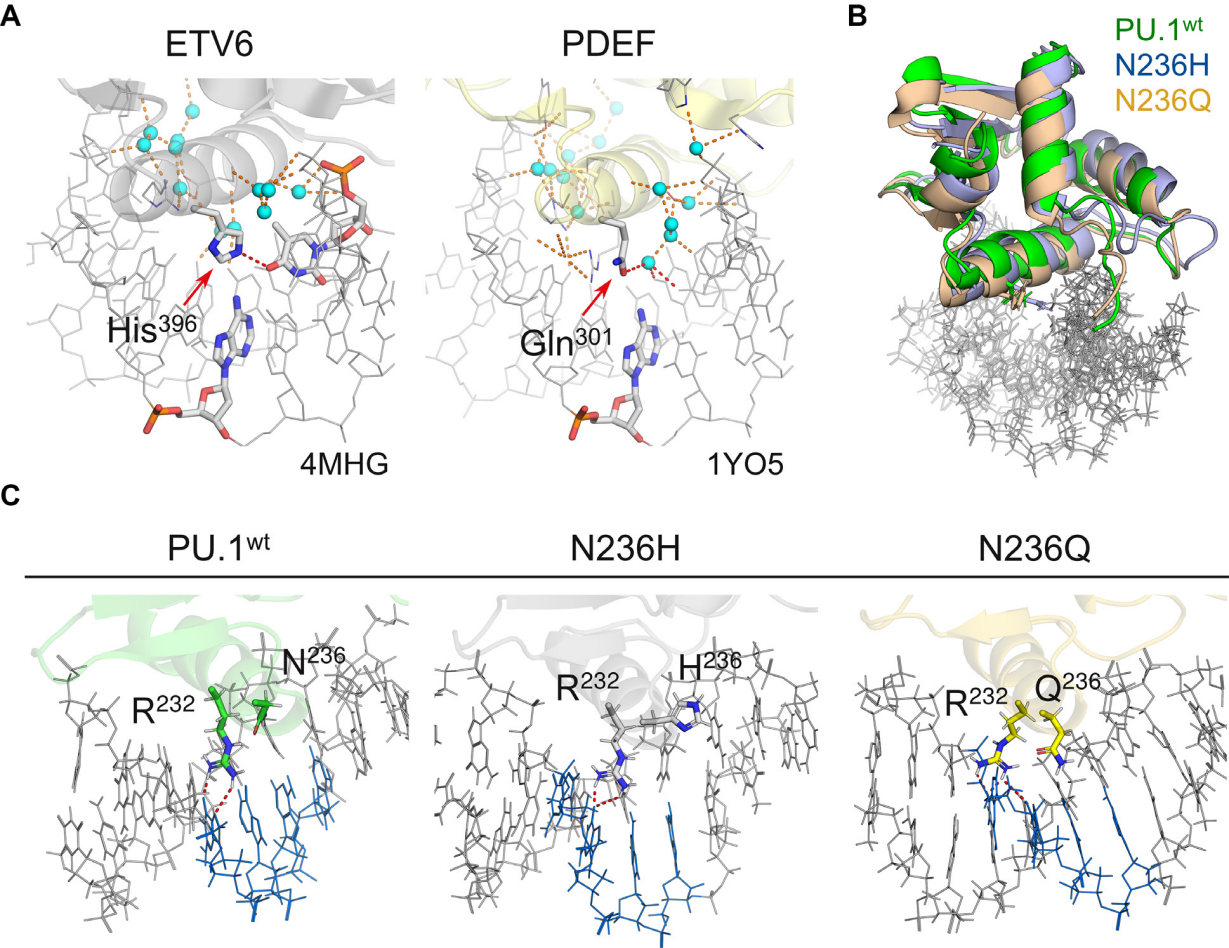
Evolutionarily conservative chimeras at Asn^236^ are also structurally conservative. (**A**) The ETS domains of ETV6 and PDEF are phylogenetic intermediates between PU.1 and Ets-1 with similar levels of co-crystallographic interfacial hydration as PU.1. Their counterparts of Asn^236^ in PU.1 are marked by arrows. (**B**) All-atom simulated complexes of N236H and N236Q with the experimental DNA target maintained the same canonical configuration as WT PU.1. As shown, the average equilibrated structures from the final 100 ns were aligned by the bound DNA only to demonstrate their configurational homology. (**C**) Average equilibrated structures of simulated complexes show the conservation of direct contacts with the 5′-GGAA-3′ consensus by the sidechain of Arg^232^.

In addition to Tyr, Asn^236^ in PU.1 are replaced by His and Gln in phylogenetic intermediates between PU.1 and Ets-1, namely ETV6, ETV7 and PDEF (Supplementary Figure S1). In the co-crystal structure of ETV6 (43), the corresponding His^396^ makes a direct H-bond not with 5′-GGAA-3′ but O4 of 5′-TTCC-3′ in the opposite strand of the core consensus (Figure 4A), with a slightly lower amount of crystallographic hydration as PU.1. In the co-crystal structure of PDEF (44), Gln^311^ makes only water-mediated contacts similarly as Asn^236^ in PU.1. Since interfacial crystallographic hydration is correlated with evolutionary separation among ETS domains (45), we asked whether substitution of Asn^236^ in PU.1 with His or Gln would be less perturbing than Tyr found in Ets-1. To address this question, we simulated DNA-bound N236H and N236Q chimeras under identical *in silico* conditions as the other complexes. Unlike N236Y, both chimeric complexes maintained the canonical complex configuration of WT PU.1 (Figure 4B) and crucially preserved direct contacts with core consensus DNA by Arg^232^ (Figure 4C; c.f. Figure 4G). Nevertheless, examination of the sidechain dihedrals showed that His^236^ had flipped out of position similarly as Tyr^236^ in N236Y, with a correspondingly similar hydration profile (Supplementary Figure S4). In N236Q, the dihedral ensemble for Gln^236^ adopted configurations that resembled those observed for Gln^301^ in the PDEF co-crystal structure. Thus, chimeric substitutions at position 236 of PU.1 with evolutionarily proximal residues were more compatible with conserving the canonical ETS/DNA complex than a more ancestral counterpart, namely Tyr found in ETS paralogs such as Ets-1.

**Figure 5.**
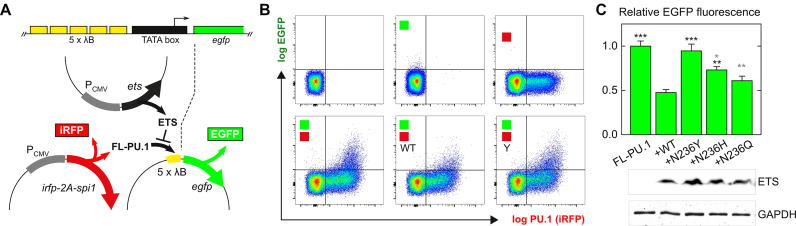
Inhibition of PU.1 transactivation by evolutionarily conservative H3 chimeras. Cognate DNA binding by PU.1 ETS mutants N236X (X = Y, H or Q) was evaluated functionally as dominant-negative inhibitors of PU.1 transactivation in HEK293 cells. (**A**) Schematic of a PU.1-dependent EGFP reporter under the control of an enhancer consisting of a 5 × tandem λB motif. Cells expressing ectopic full-length (FL) PU.1 was gated via a co-translating iRFP marker. (**B**) Representative flow cytometric data on transient HEK293 transfectants and controls. Colored squares denote co-transfection with the FL-PU.1 expression plasmid or EGFP reporter plasmid. WT and Y denote co-transfection with expression plasmid for the WT or N236Y mutant of the PU.1 ETS domain. See ‘Materials and Methods’ section for details. Axes represent logarithmic intensities of the iRFP marker and EGFP reporters. (**C**) EGFP fluorescence, adjusted for the expression of the ETS domains (by immunoblot) and normalized relative to the intensity of the no-ETS sample as mean ± S.D. (*N* = 3), and analyzed by one-way ANOVA with Tukey’s post-hoc tests. *,**,***: *p* < 0.05, 0.005, 5 × 10^−4^ (black, versus WT ETS; gray, versus N236Y).

To probe the functional significance of evolutionary variations of Asn^236^, we tested the N236Y/H/Q mutants as dominant-negative inhibitors of PU.1 transactivation in cells. We co-transfected HEK293 cells with expression plasmids encoding full-length PU.1 and WT or mutant ETS domain, together with a PU.1-dependent reporter plasmid (Figure 5A). The reporter expressed EGFP under the control of a synthetic enhancer consisting of a pentameric PU.1-binding λB motif from the Igλ_2-4_ enhancer (37). The full-length PU.1 transgene was cloned with an iRFP construct via a co-translating 2A peptide to enable isolation of PU.1-expressing cells by flow cytometry (38). In PU.1-negative HEK293 cells, the reporter was negligibly activated by endogenous transcription factors in control transfectants lacking ectopic full-length PU.1 (Figure 5B). PU.1-dependent EGFP fluorescence was measured in the subpopulation of iRFP-expressing cells (i.e. upper-right quadrant). Co-transfection of constant amounts of full-length and ETS-encoding genes showed that the N236Y mutant inhibited full-length PU.1 ineffectively relative to WT ETS domain. The N236H and N236Q mutants were both more effective inhibitors than N236Y with the N236Q being statistically similar to WT (*p* = 0.05; Figure 5C). In summary, functional studies in cells confirmed MD and biophysical characterizations that mutation of Asn^236^ perturbed DNA binding by PU.1 in an evolutionarily sensitive fashion.

### Interfacial hydration confers sequence selectivity to PU.1

**Figure 6.**
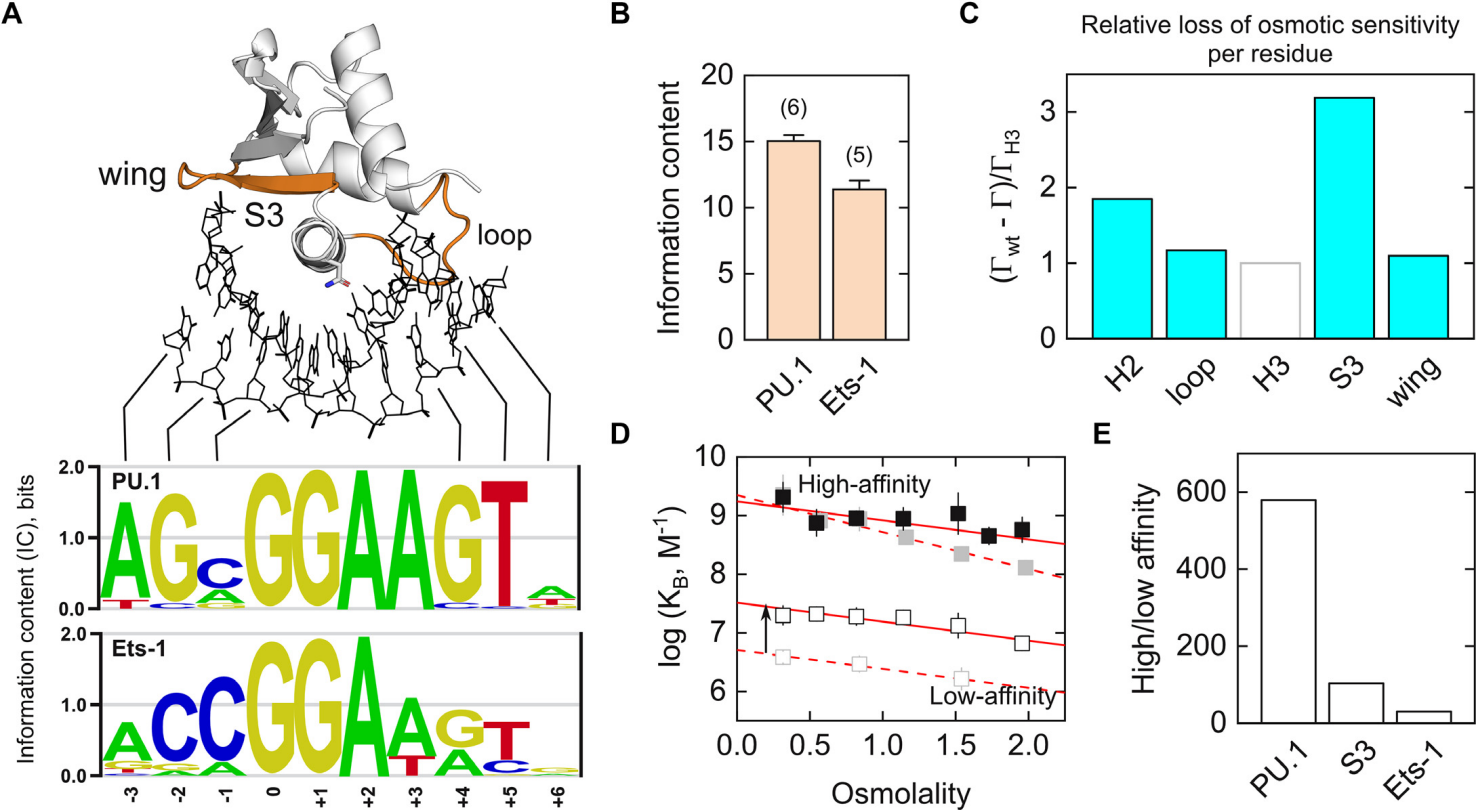
Osmotic sensitivity is associated with stringent DNA sequence discrimination by PU.1. (**A**) Positions in PU.1-binding sites with higher stringency over Ets-1 are closely contacted by structural elements that exhibit strong affinity compensation upon loss of osmotic sensitivity, colored in orange using the PU.1/DNA co-crystal structure as model. Asn^236^ is shown as sticks. The two *in vivo* sequence motifs are taken from Jolma *et al*. (48). (**B**) Average ICs ± SE of *in vivo* bound sequence motifs (10 bp) curated at the CIS-BP database (see Supplementary Methods), with the number of motifs in parenthesis. (**C**) Per-residue loss of osmotic sensitivity among the chimeras relative to WT, normalized to the H3 chimera. Only the four chimeras colored in blue exhibit affinity compensation. (**D**) Osmotic pressure dependence of high- (solid symbols) and low-affinity binding (open) by the S3 chimera (black) relative to WT PU.1 (gray; c.f. Figure 2A). (**E**) Ratio of affinities for high- versus low-affinity targets by the S3 chimera in comparison with WT PU.1 and Ets-1.

Although ETS proteins bind a spectrum of cognate DNA variants harboring a 5′-GGA(A/T)-3′, they differ in selectivity for these variants. One general approach to parameterizing sequence selectivity is to apply information theory to bound sequence motifs (46). Specifically, the information content (IC) of a sequence motif quantifies the sequence discriminating power of the ligand in terms of the number of binary bits, ranging from 0 per base position if all four bases are equally probable, to 2 if the position is fully specified by a single base (47). For PU.1 and Ets-1, we have previously determined the IC of sequence motifs from *in vitro* selection experiments as well as ChIP-Seq data on genomic binding (26). As the representative DNA logos (48) in Figure 6A show, PU.1 exerts higher selectivity at the two 5′ distally flanking bases (positions −3, −2) and four 3′ flanking bases (positions +3 to +6) over Ets-1. Relaxation of selectivity at the core +3 position from A to A/T by a N236Y mutation has been previously reported (49). The remaining base positions at which PU.1 is more selective are proximal to the wing, S3 and loop whose chimeras are strongly affinity-compensated despite significant losses of osmotic sensitivity (Table 1). An updated compilation of *in vivo* data show that over their full 10-bp binding sites (out of a maximum IC of 2 × 10 = 20 bits), PU.1 exhibits an IC that is over ∼3.5 bits or 30% higher than Ets-1 (Figure 6B). This margin is even more striking on account of the 6 bits of IC fixed by the invariant core consensus at positions 0 to +2.

The positional correlation of bases at which PU.1 is differentially selective with affinity-compensated DNA-contacting elements led us to ask whether the compensatory interactions by chimeric Ets-1 residues would be less sequence-selective than the native contacts that they replaced. To test this hypothesis, we compared the affinity of the S3 chimera, which exhibited the greatest *per-residue* loss in osmotic sensitivity (Figure 6C) and strongest affinity compensation for its optimal DNA target over an established low-affinity PU.1 binding site (14). The S3 chimera bound low-affinity DNA ∼5-fold more strongly than WT PU.1 (arrow in Figure 6D). Since both WT and S3-chimeric PU.1 bound high-affinity DNA equally well, the ratio of high/low affinity binding by S3 chimera was ∼5-fold lower than WT PU.1 (Figure 6E) and ∼3-fold higher than WT Ets-1 for its respective high- and low-affinity sequences (50). In addition, whereas the osmotic sensitivity of WT PU.1 is sequence-dependent, the S3 chimera exhibited similar osmotic sensitivity for both DNA targets, indicating a lack of hydration contribution to selectivity. Bioinformatics analysis and direct measurements therefore showed that introduction of Ets-1 residues into PU.1 reduced osmotic sensitivity and its attendant sequence selectivity.

## DISCUSSION

With the exception of H3/S3, PU.1/Ets-1 chimeras of the DNA-contact surface retain the conserved structural framework characteristic of ETS domains. The chimeric perturbations on osmotic sensitivity therefore reveal the contribution of each structural element to hydrating the DNA-binding interface. The chimeras exhibit blunted osmotic sensitivity relative to WT PU.1 roughly in step with their per-residue contact area with DNA. Overall, the data shows that hydration of the PU.1/DNA interface is not locally mediated by any one or small subset of elements and suggests a network of distributed interactions involving the contact surface as a whole. The S3 chimera is a standout in that it contributes on a per-residue basis more hydration interactions than the other DNA-contact elements.

Although all chimeras lose osmotic sensitivity, only the H3 chimera also exhibit significant loss of binding affinity in the absence of osmotic stress. The normo-osmotic affinities of the H2, loop, S3 and wing chimeras are within 5-fold of WT PU.1, which is not a significant reduction given the over 400-fold span in affinity between high- and low-affinity cognate binding (14). In previous work (30), we reported that disruption of a crystallographic water contact by the interfacial mutation Y252F in PU.1 reduced osmotic sensitivity by ∼25%, but without effect on normo-osmotic binding affinity to the same high-affinity DNA used here. The chimeric data for H2, loop, S3 and wing therefore reinforces and generalizes the concept of affinity compensation. As osmotic stress raises the free energy cost of accumulating interfacial water, the decoupling of osmotic sensitivity and binding affinity indicates that the loss of favorable hydration contributions in these chimeras can be offset by Ets-1 residues to achieve almost the same affinity. The compatibility of alternative interactions with high-affinity binding over large portions of the DNA-contact surface reflects the low primary structure conservation of these domains among paralogous ETS proteins.

An important functional consequence of hydration in DNA recognition by PU.1 is that relative to a sparsely hydrated relative Ets-1, the incorporation of water molecules increases the sequence selectivity of binding. The sequence motifs bound by the two native ETS paralogs show deficits in IC for Ets-1 at most base positions flanking the 5′-GGA(A/T)-3′ central consensus. These flanking bases are most closely contacted by DNA-contacting elements whose Ets-1 chimeras exhibit the highest reduction of osmotic sensitivity with affinity compensation. As demonstrated by the S3 chimera, substitution with Ets-1 residues results in a strongly affinity-compensated species with increased affinity for a low-affinity target, narrowing the selectivity gap between optimal and low-affinity DNA. Moreover, the S3 chimera shows no hydration contribution to selectivity, in contrast to the strong sequence dependence in osmotic sensitivity found in WT PU.1. Taken together, the data supports the hypothesis that divergent sequences of PU.1 and Ets-1 are linked to greater target selectivity for PU.1.

### Significance of the H3 recognition helix in the ETS motif

Unlike the other well-folded chimeras, H3 exhibits significant loss of normo-osmotic binding as well as sensitivity to osmotic pressure. The PU.1-specific H3 residues are therefore highly adapted to the other elements of the PU.1 ETS domain. Of all the secondary structures in the ETS domain, H3 exposes the largest fraction of its surface area to the DNA contact interface. As shown by the N236Y mutant, which recapitulates the binding properties of the full H3 chimera, the canonical ETS/DNA complex configuration is disrupted by a large re-orientation of the protein relative to the target DNA site. In agreement with this structural prediction, functional assays using a PU.1-dependent EGFP reporter confirmed that the N236Y chimera exhibited significantly impaired DNA binding in live cells. H3 is also the most conserved element in the ETS domain (Supplementary Figure S1). Residue 236 is the only H3 position that exhibits significant variation: Asn, Tyr, His, Gln. On the one hand, all four residues are related by exactly a single transition in their codons (A↔G or C↔T) and align with the overall phylogenetic separation among ETS paralogs (19). On the other hand, the relative compatibility of these residues with the PU.1 scaffold in both MD and reporter assays correspond to their relative hydrophobicity in the order Tyr > His > Gln > Asn at near-neutral pH (51). Thus, the significance of H3 in PU.1/DNA binding is also closely tied to interfacial hydration and suggests an evolutionary transition in the essential recognition helix toward the acquisition of interfacial hydration.

### The structure and thermodynamics of interfacial hydration in ETS/DNA complexes

To date, structures of site-specific DNA complexes for approximately half of the 28 human and mouse ETS domains have been solved crystallographically. At comparable resolutions and irrespective of binding partners, PU.1/DNA complexes harbor more than twice as many bridging water molecules in its interface relative to Ets-1/DNA complexes (Supplementary Figure S5). Many of the corresponding residues in Ets-1 make direct DNA contacts instead. The excess bridging water in PU.1 is found all along its DNA interface, consistent with the distributed pattern reported by osmotic stress. In addition to ordered bridging water, more dynamic and weakly held water that is nevertheless energetically important do not appear in the structures (52). The global hydration change due to DNA binding is detected thermodynamically as the osmotic pressure dependence of the binding affinity via Equation (2). High-affinity binding by WT PU.1 is associated with the net uptake of ∼90 water molecules, while Ets-1 binding is net hydration-neutral (c.f. Figure 2A). From the PU.1/DNA co-crystal structure, the total water-accessible surface area of the protein/DNA interface is 985 ± 24 Å² (average of the two asymmetric units in 1PUE). Taking the nominal cross-sectional area of a water molecule as 9 Å², the osmotic sensitivity of WT PU.1 implies that up to $\frac{{90\,{\rm{water}} \times {9}\, {\raise0.5ex\hbox{$\scriptstyle {{\rm\AA}}$} \kern-0.1em/\kern-0.15em \lower0.25ex\hbox{$\scriptstyle {{\rm{water}}}$}}}}{{985\,{{\rm\AA}^2}}} = \sim 80\%$ of the its interface with DNA becomes net hydrated. Comparison of this total hydration change with the co-crystal structure therefore identifies distinct populations of hydration water. Beyond the ∼15 ordered bridging hydration as captured crystallographically, an additional number of more dynamic water molecules are involved in hydrating the PU.1/DNA complex. Correspondingly, a quantity of weakly held water is net displaced upon formation of the Ets-1/DNA complex.

In addition to affinity, which corresponds to the free energy change, the hydration contributions to DNA binding by PU.1 and Ets-1 are manifest in the underlying thermodynamic parameters. High-affinity PU.1 binding exhibits an unusually small negative change in heat capacity (53,54), a parameter for which large magnitudes are interpreted as dehydration (release of hydration water) upon binding (55). Subsequent calorimetric comparisons of ETS domains from WT PU.1 and Ets-1 show that binding is entropically more favorable for Ets-1 than PU.1 and accompanied by a more negative heat capacity change (25). These thermodynamic signatures are consistent with a configurational penalty paid by dynamically restricted water in the PU.1/DNA interface. Osmotically blunted but affinity-compensated PU.1/Ets-1 chimeras are therefore expected to be more entropically driven with more negative changes in heat capacity, effectively tending toward the thermodynamic profile of WT Ets-1.

### CONCLUSION

The ETS-family transcription factors PU.1 and Ets-1 bind their respective cognate DNA targets with similarly high affinity under normo-osmotic conditions but exhibit profound differences in their sensitivity to osmotic pressure. By interrogating chimeric ETS domains consisting of targeted substitutions of Ets-1 residues into PU.1, the present data squarely implicate interfacial hydration as a specificity determinant in DNA recognition by PU.1. More generally, the relationship between affinity and specificity continues to be a matter of fundamental interest in protein/DNA interactions. The structurally conserved ETS factors offer a biological venue in which this question can be addressed, while also providing insight into the likely functional roles of these specific transcription factors. Given the prominent role of ETS domains in directing the molecular properties of the full-length proteins, this study also supports chimeras as a rational approach to dissect other functional and evolutionary relationships of ETS transcription factors.

## Supplementary Material

Supplementary Data
