# Supplementary material for: Mapping interfacial hydration in ETS-family transcription factor complexes with DNA: a chimeric approach
Source: Nucleic Acids Res. 2018 Oct 8;46(20):10577–88. doi: 10.1093/nar/gky894 (PMC6237740; doi:10.1093/nar/gky894)
Supplement: Supplementary Data [file gky894_supplemental_files.pdf]

SUPPLEMENTAL INFORMATION

# Mapping interfacial hydration in ETS-family transcription factor complexes with DNA: a chimeric approach

September 16, 2018

Amanda V. Albrecht,[1,*] Hye Mi Kim,[1,*] and Gregory M. K. Poon[1,2,**]

[1] Department of Chemistry, Georgia State University, Atlanta, GA 30303

[2] Center for Diagnostics and Therapeutics, Georgia State University, Atlanta, GA 30303

** These authors contributed equally to this study.

* To whom correspondence should be addressed at: P.O. Box 3965, Atlanta, GA 30302-3965. Email: gpoon@gsu.edu. Tel. (404) 413-5491. Fax. (404) 413-5505.

## SUPPLEMENTAL METHODS

*Analysis of PU.1/DNA titration data.* The effect of osmolytes on DNA recognition, when extrinsically labeled DNA is used as probe, is not accurately reported by direct binding to the probe DNA due to extraneous interactions by the osmolyte with the fluorescent label (1). We therefore measure the affinity of PU.1 with *unlabeled* DNA indirectly via its inhibition of the PU.1/probe complex. Titration of Cy3-labeled DNA and PU.1 with unlabeled DNA is described by a competitive model in which PU.1 (denoted 01) binds either the labeled probe (1*0; the asterisk denotes probe) or unlabeled competitor (10), but not both, to form the complex 1*1 or 11 respectively. We previously applied this model to describe the competition of PU.1 and other ETS proteins between probe and various unlabeled DNA competitors (2-4). The binding polynomial, which is cubic in [11] is,

$$0 = \varphi_0 + \varphi_1[11] + \varphi_2[11]^2 + \varphi_3[11]^3$$
$$\begin{cases} \varphi_0 = -K_{1*1}[10]_t^{\,2}[01]_t \\ \varphi_1 = K_{11}K_{1*1}[10]_t + K_{11}[1*0]_t[10]_t + K_{1*1}[10]_t^{\,2} + 2K_{1*1}[10]_t[01]_t - K_{11}[10]_t[01]_t \\ \varphi_2 = -K_{11}K_{1*1} + K_{11}^{\,2} - K_{11}[1*0]_t - 2K_{1*1}[10]_t + K_{11}[10]_t - K_{1*1}[01]_t + K_{11}[01]_t \\ \varphi_3 = K_{1*1} - K_{11} \end{cases} \quad \text{(S1)}$$

where $K_{1*1} = \frac{[1*0][01]}{[1*1]}$ and $K_{11} = \frac{[10][01]}{[11]}$ are equilibrium dissociation constants for the labeled and unlabeled complex. This mechanistic model therefore directly estimates binding affinities and completely separates any osmolyte-specific effects on probe binding. Note also that the model does not use of the $IC_{50}$ (the so-called Cheng-Prusoff procedure; 5). $F_b = \frac{[11]}{[11]_t}$ is substituted into Eq. (1) in the main text to fit the concentration-dependent anisotropy $\langle r \rangle$. $K_{11}$ (for the unlabeled

DNA) is the equilibrium constant of interest reported in the main text. Typically, sub-saturating concentrations of PU.1 (in the absence of competitor) are used to relieve depletion effects that tend to introduce sharp curvature into the data (6). $K_{1*1}$ can be independently determined from direct titration of the probe with protein alone.

*Bioinformatics analysis of sequence motifs*. Curated sequence motifs for human PU.1 (SPI1) and Ets-1 derived from ChIP-Seq analysis were culled from the CIS-BP database (7). Motifs that did not cover the a 10-bp window centered at the 5'-GGA(A/T)-3' consensus were excluded. The position frequency matrix of each motif was analyzed by enoLOGOS (8) to extract the information content (IC) at each position and to generate a sequence logo. No species-related bias in GC content was included in the IC computations (i.e., each base was assumed to be equiprobable).

## INDEX TO SUPPLEMENTAL TABLE AND FIGURES


**Table S1** MALDI-ToF analysis of wildtype and chimeric ETS domains of PU.1

**Figure S1** Multiple sequence alignment of the murine ETS-family of transcription factors

**Figure S2** Thermal unfolding and refolding of PU.1/Ets-1 chimeras

**Figure S3** The ETS domain of wildtype Ets-1 binds optimal cognate DNA target for PU.1 with similar affinity under physiologic conditions in vitro

**Figure S4** Standard sidechain dihedrals and H-bonding of the chimeric residue in simulated N236H and N236Q mutants of the PU.1 ETS domain

**Figure S5** Crystallographic interfacial water in PU.1 and Ets-1 complexes with high-affinity DNA

**Table S1**

*MALDI-ToF analysis of wildtype and chimeric ETS domains of PU.1*

Ten ng of purified recombinant protein was examined by MALDI-ToF(+) mass spectroscopy. In all cases, two *m/z* peaks corresponding to the +1 (base peak) and +2 molecular ions were observed. The expected molecular weights were computed based on elements at natural isotopic abundance and *in situ* cleavage of the leading methionine by bacterial aminopeptidase (4).

| | Molecular weight, Da | | |
|---|---|---|---|
| | *Expected (E)* | *Observed (O)* | %(*O* – *E*) |
| *Wildtype* | 12,847 | 12,844 | -0.02 |
| *Chimeras* | | | |
| H2 | 12,843 | 12,818 | -0.2 |
| Loop | 12,774 | 12,784 | +0.08 |
| H3 | 12,894 | 12,898 | +0.03 |
| H3/S3 | 13,024 | 13,030 | +0.05 |
| S3 | 12,927 | 12,957 | +0.2 |
| Wing | 12,875 | 12,879 | +0.03 |
| N236Y | 12,455 | 12,459 | +0.03 |

```
sp|Q6P3D7|112-195|SPIC|Mus_musculus           LRLFEYLFESLCNS-EMVSCIQWVDKARGIFQFISKNKETLAELWGQRKGNRKPMTYQKMARALRNYARTGEIIKIR--RKLTYQFS   84
sp|P17433|172-255|SPI1|Mus_musculus           IRLYQFLLDLLRSG-DMKDSIWWVDKDKGTFQFSSKHKEALAHRWGIQKGNRKKMTYQKMARALRNYGKTGEVKKVK--KKLTYQFS   84
sp|O35906|174-257|SPIB|Mus_musculus           LRLYQFLLGLLLRG-DMRECVWWVEPGAGVFQFSSKHKELLARRWGQQKGNRKRMTYQKLARALRNYAKTGEIRKVK--RKLTYQFD   84
sp|Q9WTP3|239-322|SPDEF|Mus_musculus          IHLWQFLKELLLKPHSYGRFIRWLNKEKGIFKIED--SAQVARLWGVRK-NRPAMNYDKLSRSIRQYYKKGIIRKPDISQRLVYQFV   84
sp|Q60775|208-290|ELF1|Mus_musculus           IYLWEFLLALLQDKATCPKYIKWTQREKGIFKLVD--SKAVSRLWGKHK-NKPDMNYETMGRALRYYYQRGILAKVE-GQRLVYQFK   83
sp|Q9Z2U4|208-290|ELF4|Mus_musculus           IYLWEFLLALLQDRNTCPKYIKWTQREKGIFKLVD--SKAVSKLWGKQK-NKPDMNYETMGRALRYYYQRGILAKVE-GQRLVYQFK   83
sp|Q9JHC9|208-290|ELF2|Mus_musculus           TYLWEFLLDLLQDKNTCPRYIKWTQREKGIFKLVD--SKAVSKLWGKHK-NKPDMNYETMGRALRYYYQRGILAKVE-GQRLVYQFK   83
sp|Q8VDK3|161-242|ELF5|Mus_musculus           SHLWEFVRDLLLSPEENCGILEWEDREQGIFRVVK--SEALAKMWGQRK-KNDRMTYEKLSRALRYYYKTGILERVD--RRLVYKFG   82
sp|O70273|207-289|EHF|Mus_musculus            THLWEFIRDILLSPDKNPGLIKWEDRSEGIFRFLK--SEAVAQLWGKKK-NNSSMTYEKLSRAMRYYYKREILERVD-GRRLVYKFG   83
sp|Q3UPW2|293-375|ELF3|Mus_musculus           THLWEFIRDILIHPELNEGLMKWENRHEGVFKFLR--SEAVAQLWGQKK-KNSNMTYEKLSRAMRYYYKREILERVD-GRRLVYKFG   83
sp|P97360|335-416|ETV6|Mus_musculus           RLLWDYVYQLLSDS-RYENFIRWEDKESKIFRIVD--PNGLARLWGNHK-NRTNMTYEKMSRALRHYYKLNIIRKEP-GQRLLFRFM   82
tr|G1TVM5|311-392|ETV7|Oryctolagus_cuniculus  RLLWDFVYQLLLDP-RYEPYIRWEDKDAKIFRVVD--PNGLAGLWGKHK-NRVNMTYEKLSRALRHYYKLNIIRKEP-GQKLVFRFL   82
sp|P41969|5-86|ELK1|Mus_musculus              VTLWQFLLQLLREQ-GNGHIISWTSRDGGEFKLVD--AEEVARLWGLRK-NKTNMNYDKLSRALRYYYDKNIIRKVS-GQKFVYKFV   82
sp|P41971|5-85|ELK3|Mus_musculus              ITLWQFLLHLLLDQ-KHEHLICWTSND-GEFKLLK--AEEVAKLWGLRK-NKTNMNYDKLSRALRYYYDKNIIKKVI-GQKFVYKFV   81
sp|P41158|5-85|ELK4|Mus_musculus              ITLWQFLLQLLQEP-QNEHMICWTSNN-GEFKLLQ--AEEVARLWGIRK-NKPNMNYDKLSRALRYYYVKNIIKKVN-GQKFVYKFV   81
sp|P70459|27-107|ERF|Mus_musculus             IQLWHFILELLRKE-EYQGVIAWQ-GDYGEFVIKD--PDEVARLWGVRK-CKPQMNYDKLSRALRYYYNKRILHKTK-GKRFTYKFN   81
sp|Q8R4Z4|35-116|ETV3|Mus_musculus            IQLWHFILELLQKE-EFRHVIAWQQGEYGEFVIKD--PDEVARLWGRRK-CKPQMNYDKLSRALRYYYNKRILHKTK-GKRFTYKFN   82
tr|A0JPP2|40-121|ETV3L|Rattus_norvegicus      IQLWHFILELLQKE-EFRHVIAWQQGEYGEFVIKD--PDEVARLWGRRK-CKPQMNYDKLSRALRYYYNKRILHKTK-GKRFTYKFN   82
sp|P41164|335-415|ETV1|Mus_musculus           LQLWQFLVALLDDP-SNSHFIAWT-GRGMEFKLIE--PEEVARRWGIQK-NRPAMNYDKLSRSLRYYYEKGIMQKVA-GERYVYKFV   81
sp|P28322|342-422|ETV4|Mus_musculus           LQLWQFLVALLDDP-TNAHFIAWT-GRGMEFKLIE--PEEVARLWGIQK-NRPAMNYDKLSRSLRYYYEKGIMQKVA-GERYVYKFV   81
sp|Q9CXC9|368-448|ETV5|Mus_musculus           LQLWQFLVTLLDDP-ANAHFIAWT-GRGMEFKLIE--PEEVARRWGIQK-NRPAMNYDKLSRSLRYYYEKGIMQKVA-GERYVYKFV   81
sp|P41163|234-314|ETV2|Mus_musculus           IQLWQFLLELLHDG-ARSSCIRWT-GNSREFQLCD--PKEVARLWGERK-RKPGMNYEKLSRGLRYYYRRDIVLKSG-GRKYTYRFG   81
sp|Q00422|320-400|GABPA|Mus_musculus          IQLWQFLLELLTDK-DARDCISWV-GDEGEFKLNQ--PELVAQKWGQRK-NKPTMNYEKLSRALRYYYDGDMICKVQ-GKRFVYKFV   81
sp|P27577|335-415|ETS1|Mus_musculus           IQLWQFLLELLTDK-SCQSFISWT-GDGWEFKLSD--PDEVARRWGKRK-NKPKMNYEKLSRGLRYYYDKNIIHKTA-GKRYVYRFV   81
sp|P15037|362-442|ETS2|Mus_musculus           IQLWQFLLELLSDK-SCQSFISWT-GDGWEFKLAD--PDEVARRWGKRK-NKPKMNYEKLSRGLRYYYDKNIIHKTS-GKRYVYRFV   81
sp|Q8QZW2|47-127|FEV|Mus_musculus             IQLWQFLLELLADR-ANAGCIAWE-GGHGEFKLTD--PDEVARRWGERK-SKPNMNYDKLSRALRYYYDKNIMSKVH-GKRYAYRFD   81
sp|P81270|318-398|ERG|Mus_musculus            IQLWQFLLELLSDS-SNSNCITWE-GTNGEFKMTD--PDEVARRWGERK-SKPNMNYDKLSRALRYYYDKNIMTKVH-GKRYAYKFD   81
sp|P26323|281-361|FLI1|Mus_musculus           IQLWQFLLELLSDS-ANASCITWE-GTNGEFKMTD--PDEVARRWGERK-SKPNMNYDKLSRALRYYYDKNIMTKVH-GKRYAYKFD   81
                                                *:.::   *         : *       * .       ::  ** :*  .  *.*:.:.*.:* *     : :    .:  ::*
```

**Figure S1. Multiple sequence alignment of the murine ETS-family of transcription factors.** Sequences were culled from UniProt and aligned by Clustal Omega, using the house mouse (*M. musculus*) as reference organism. In two cases (ETV7 and ETV3L) where an annotated paralog is not available, we used the ortholog from rabbit (*O. cuniculus*) and rat (*R. norvegicus*). PU.1 (Spi-1) and Ets-1 are in black. The orthologous residues (Tyr, His, and Gln) corresponding to $Asn^{236}$ in PU.1 are colored in red.

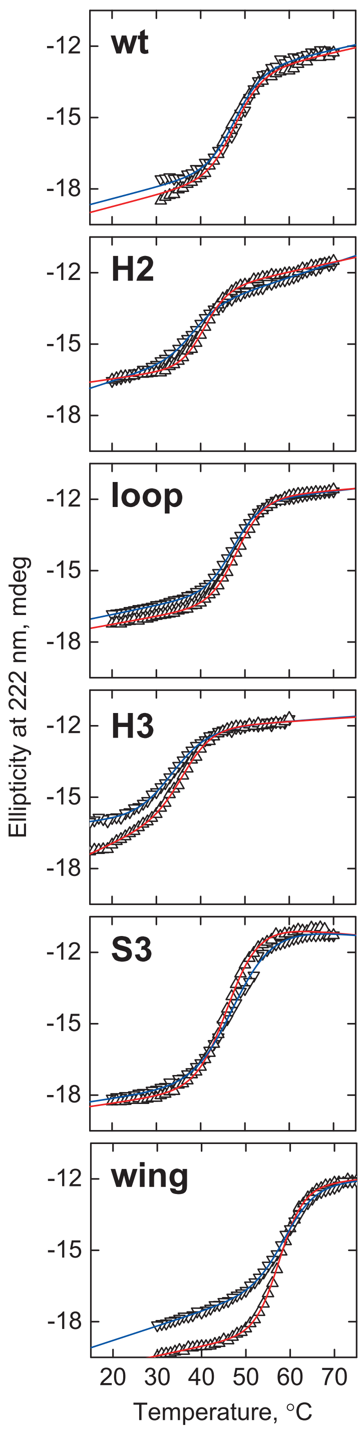


Figure S2. **Thermal unfolding and refolding of PU.1/Ets-1 chimeras.** CD-monitored (at 222 nm) melting (△) and refolding (▽) was carried out with 25 μM wildtype and chimeric PU.1 ETS domains under normo-osmotic conditions at ±45°C/h and a response time of 32 s. The H3/S3 chimera, which did not fold natively as PU.1, was not tested. *Curves* represent fits to the unfolding/refolding data (red/blue) by a two-state transition (9). The averaged melting temperature ($T_m$) of the heating and cooling runs were reported ±1°C in Figure 2d of the main text.

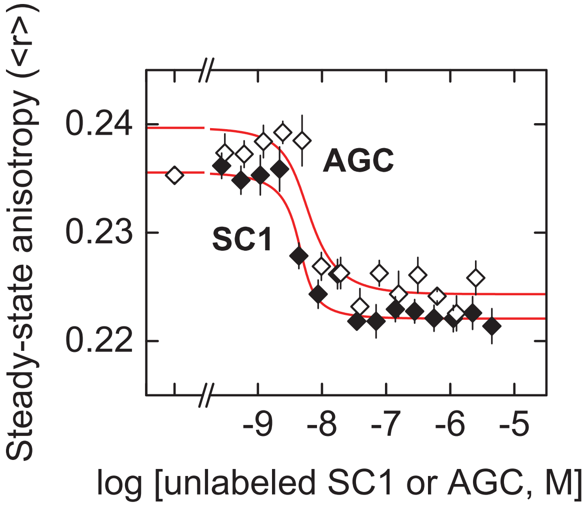


Figure S3. **The ETS domain of wildtype Ets-1 binds optimal cognate DNA target for PU.1 with similar affinity under physiologic conditions in vitro.** The normo-osmotic affinity of the C-terminal ETS domain of wildtype Ets-1 (murine residues 331 to 440, without the auto-inhibitory helices) for its optimal DNA (SC1: 5'-GCCGGAAGTG-3') and optimal DNA for PU.1 (AGC: 5'-AGCGGAAGTG-3') was determined by fluorescence polarization exactly as described in the main text. The affinities for SC1 and AGC are 0.17 ± 0.14 nM and 0.6 ± 0.3 nM, respectively.

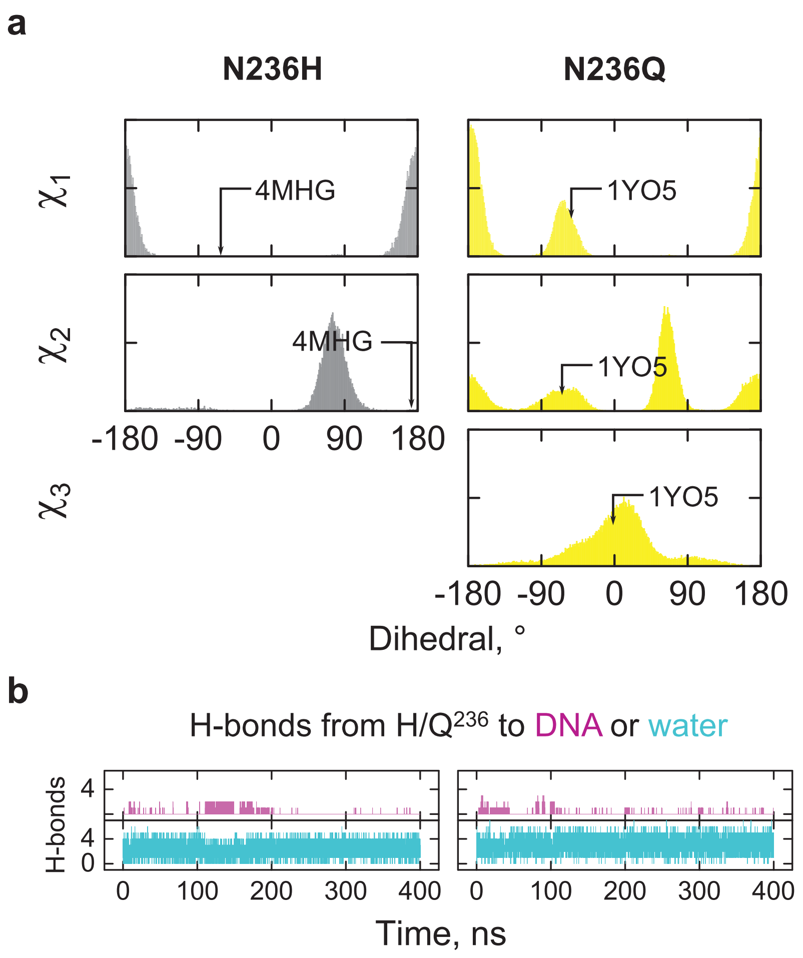


Figure S4. **Standard sidechain dihedrals and H-bonding of the chimeric residue in simulated N236H and N236Q mutants of the PU.1 ETS domain. a**, Equilibrated trajectories from the final 100 ns of simulation were analyzed as for Figure 4f in the main text. *Arrows* mark the dihedrals from the co-crystal structures of ETV6 (4MHG; 10) and SPDEF (1YO5; 11), two proximal ETS relatives that harbor His and Gln at the corresponding positions as $Asn^{236}$ in wildtype PU.1. **b**, H-bond contacts (<3.5 Å and ±30° between heavy donor/acceptor pairs) formed by the chimeric residue with DNA and water. Note the similar burst of direct DNA contacts formed by $His^{236}$ in N236H between 100 to 200 ns as $Tyr^{236}$ in N236Y (Figure 4d in the main text), but is absent in wildtype or N236Q.

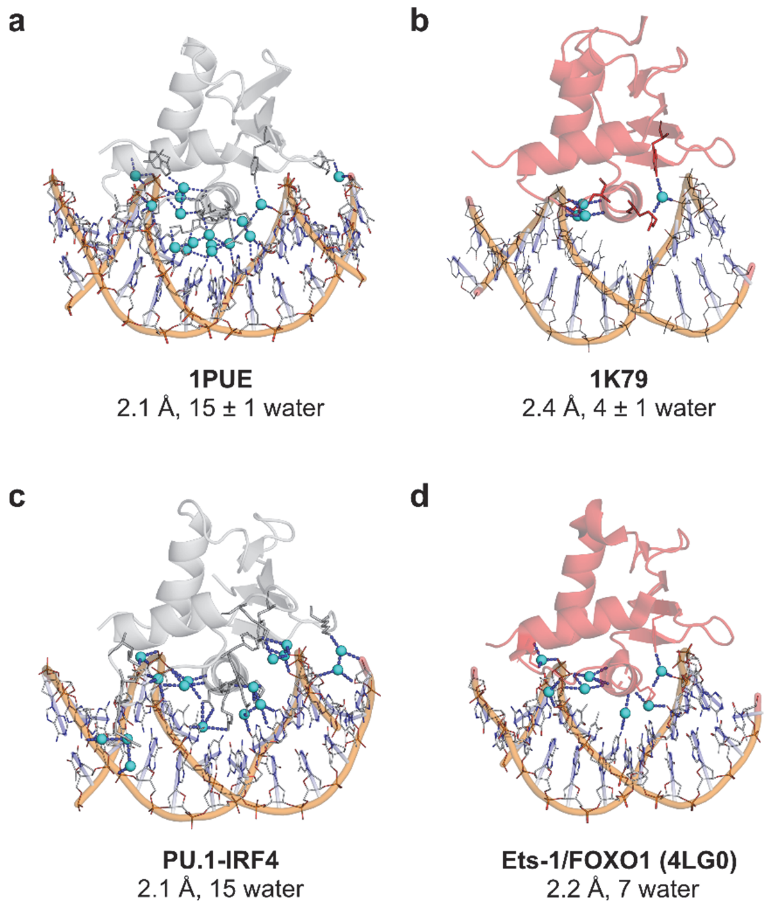


Figure S5. **Crystallographic interfacial water in PU.1 and Ets-1 complexes with high-affinity DNA.** Only bridging waters within 3.5 Å between donor/acceptor are enumerated; non-bridging crystallographic hydration (whose disposition in the unbound states cannot be inferred only from the complex) is excluded. The refinement resolutions are given. **a and b**, Binary ETS/DNA structures of the PU.1 (1PUE) and Ets-1 (1K79). These co-crystals contain two asymmetric units per unit cell. The bridging water in both asymmetric units is counted and averaged. **c and d**, PU.1 and Ets-1 in ternary structures with a binding partner: IRF4 for PU.1 (12), FOXO1 for Ets-1. The binding partner is not rendered. These structures have only one asymmetric unit per unit cell.

## SUPPLEMENTAL REFERENCES


1. Stephens, D.C., Kim, H.M., Kumar, A., Farahat, A.A., Boykin, D.W. and Poon, G.M.K. (2016) Pharmacologic efficacy of PU.1 inhibition by heterocyclic dications: a mechanistic analysis. *Nucleic Acids Res*, **44**, 4005-4013.
2. Poon, G.M. (2012) Sequence discrimination by DNA-binding domain of ETS family transcription factor PU.1 is linked to specific hydration of protein-DNA interface. *J Biol Chem*, **287**, 18297-18307.
3. Wang, S., Linde, M.H., Munde, M., Carvalho, V.D., Wilson, W.D. and Poon, G.M. (2014) Mechanistic heterogeneity in site recognition by the structurally homologous DNA-binding domains of the ETS family transcription factors Ets-1 and PU.1. *J Biol Chem*, **289**, 21605-21616.
4. Stephens, D.C. and Poon, G.M. (2016) Differential sensitivity to methylated DNA by ETS-family transcription factors is intrinsically encoded in their DNA-binding domains. *Nucleic Acids Res*, **44**, 8671-8681.
5. Yung-Chi, C. and Prusoff, W.H. (1973) Relationship between the inhibition constant (KI) and the concentration of inhibitor which causes 50 per cent inhibition (I50) of an enzymatic reaction. *Biochem Pharmacol*, **22**, 3099-3108.
6. Wells, J.W. (1992) In Hulme, E. C. (ed.), *Receptor-Ligand Interactions: a Practical Approach*. IRL Press at Oxford University Press, Oxford [England]; New York, pp. 289-395.
7. Weirauch, M.T., Yang, A., Albu, M., Cote, A.G., Montenegro-Montero, A., Drewe, P., Najafabadi, H.S., Lambert, S.A., Mann, I., Cook, K. *et al.* (2014) Determination and inference of eukaryotic transcription factor sequence specificity. *Cell*, **158**, 1431-1443.

8. Workman, C.T., Yin, Y., Corcoran, D.L., Ideker, T., Stormo, G.D. and Benos, P.V. (2005) enoLOGOS: a versatile web tool for energy normalized sequence logos. *Nucleic Acids Res*, **33**, W389-W392.

9. Poon, G.M., Groß, P. and Macgregor, R.B., Jr. (2002) The sequence-specific association of the ETS domain of murine PU.1 with DNA exhibits unusual energetics. *Biochemistry*, **41**, 2361-2371.

10. De, S., Chan, A.C., Coyne, H.J., 3rd, Bhachech, N., Hermsdorf, U., Okon, M., Murphy, M.E., Graves, B.J. and McIntosh, L.P. (2014) Steric mechanism of auto-inhibitory regulation of specific and non-specific DNA binding by the ETS transcriptional repressor ETV6. *J Mol Biol*, **426**, 1390-1406.

11. Wang, Y., Feng, L., Said, M., Balderman, S., Fayazi, Z., Liu, Y., Ghosh, D. and Gulick, A.M. (2005) Analysis of the 2.0 A crystal structure of the protein-DNA complex of the human PDEF Ets domain bound to the prostate specific antigen regulatory site. *Biochemistry*, **44**, 7095-7106.

12. Escalante, C.R., Brass, A.L., Pongubala, J.M., Shatova, E., Shen, L., Singh, H. and Aggarwal, A.K. (2002) Crystal structure of PU.1/IRF-4/DNA ternary complex. *Mol Cell*, **10**, 1097-1105.
